# Appraisal of interpretation criteria for the single intra-dermal comparative cervical tuberculin test for the diagnosis of tuberculosis in dromedary camels in Ethiopia

**DOI:** 10.1007/s11250-018-1610-y

**Published:** 2018-05-02

**Authors:** Yasmin Jibril, Gezahegne Mamo, Ahmed Issa, Aboma Zewude, Gobena Ameni

**Affiliations:** 1 Department of Clinical Studies, College of Veterinary Medicine and Agriculture, Addis Ababa University, P.O.Box 34, Bishoftu, Ethiopia; 2 Department of Microbiology, Immunology and Veterinary Public Health, College of Veterinary Medicine and Agriculture, Addis Ababa University, P.O.Box 34, Bishoftu, Ethiopia; 3 Aklilu Lemma Institute of Patho-Biology, Addis Ababa University, P.O.Box 1176, Addis Ababa, Ethiopia

**Keywords:** Cutoff value, Dromedary camel, Gross pathology, Tuberculosis, Tuberculin skin test, Ethiopia

## Abstract

Dromedary camels are the main sources of milk, meat and income for the Ethiopian pastoralists as they withstand the harsh environments of the regions of the country. Tuberculosis (TB) affects dromedary camels causing morbidity and mortality in these animals. Hence, early diagnosis and identification of infected camels play a significant role in reducing the transmission of TB in camels. This study was conducted on 168 camels between October 2014 and July 2015 to evaluate the performance of single intra-dermal comparative cervical tuberculin (SICCT) to diagnose TB in camels. Gross pathology was used as a gold standard to define disease status of each camel. The result showed that at the cutoff value of ≥ 3 mm SICCT had optimum performance with sensitivity and specificity of 60.7 and 85%, respectively. Moreover, at a cutoff ≥ 3 mm, the receiver operating characteristics (ROC) revealed area under the ROC curve was 0.729 (0.615–0.842) which is statistically significant (*p* = 0.000). Thus, the result of the present study could suggest the use of ≥ 3 mm cutoff value for the diagnosis of TB in dromedary camels in Ethiopia.

## Introduction

The one-humped camel, dromedary (*Camelus dromedarius*), is one of the world’s hardiest domesticated animals; a vital source of transport, meat, milk, and income for pastoralists in the Sahel, East Africa, the Middle East, and South Asia. Tuberculosis (TB) is an infectious, chronic, and granulomatous disease caused by mycobacterial species belonging to the Mycobacterium tuberculosis complex (MTBC) (Thoen 2009). It affects many vertebrate animals and manifests particularly in the lungs and lymph nodes including other organs (Thoen 2009; WHO 2015). Camelids were not considered highly susceptible to TB (Fowler 2010) but in recent years, serious concerns have arisen about TB in new world camelids (NWC) particularly in LLama and alpacas in some countries where they are reared (Twomey et al. 2010). Tuberculosis also affects old world camelids (OWC) including Dromedaries and Bactrian camels (Mustafa 2013; Rhodes et al. 2015). There is little published information on the epidemiology of TB specifically relating to camelids (Rhodes et al. 2015). However, recently, few studies have been conducted on the epidemiology of tuberculosis in camels (Beye et al. 2014; Gumi et al. 2012; Jibril et al. 2016; Kassaye et al. 2013; Mamo et al. 2009, 2011; Zerom et al. 2013). Prevalence data on camel tuberculosis reported in Ethiopia showed lesion prevalence of 5.07 and 10.04% by Mamo et al. (2009, 2011); and 4.52% by (Kassaye et al. 2013). Based on comparative tuberculin test, prevalence of 9.82% at a cutoff ≥ 4 mm and 17.05% at a cutoff ≥ 2 mm were recorded (Jibril et al. 2016). Moreover, prevalence of 8.3% on post mortem examination and tuberculin reactor prevalence of 6.0% at cutoff > 4 mm (Beye et al. 2014).

Intra-dermal tuberculin skin test is the international choice method for field diagnosis of bovine TB in live animals and the World Organization for Animal Health (OIE) recommended difference between the increases in skin thickness for the test to be positive should be at least 4 mm after 72 h (OIE 2009). However, the performance of TST is affected by environmental and host factors; and the nature of the tuberculin used (Ameni et al. 2008; De la Rua-Domenech et al. 2006). In Ethiopia, pastoralist areas are well known for high TB prevalence where the pastoralists keep large number of livestock as a means of livelihood and survival strategy in the arid and semi-arid regions of the country (Rhodes et al. 2015). Moreover, pastoralists have the practice of branding camels against the prevailing tropical diseases that might have an influence on tuberculin skin reaction. A perfect cutoff point in a specific geographic area or country may not be useful in another environment or country and the ability of the test to accurately predict true positive disease status depends on its sensitivity, specificity and prevalence of the disease in the population tested (De la Rua-Domenech et al. 2006; Strain et al. 2011). Although the official TB screening method for camelids traded internationally is the tuberculin skin test (TST), none of the tests currently available can diagnose TB in camelids with certainty as none has been properly validated in these animal species (OIE 2008). Therefore, this study was aimed with the objective to assess the performance of the SICCT at different cutoff points against gross pathology for tuberculosis lesion as gold standard.

## Mathodology

### Study area

This study was conducted from October, 2014 to July, 2015 to investigate the performance of the SICCT to diagnose tuberculosis in dromedary camels slaughtered at Akaki abattoir, Central Ethiopia. Ethiopia is a country with different agro-ecology and climatic conditions that might influence the distribution of diseases and related attributes. Pastoralist camels are reared in hot and arid climatic conditions of the low land areas with extensive livestock production system. Akaki town is one of the districts of Addis Ababa, the capital city, located in the southern part of Addis Ababa at a latitude and longitude range of 8^°^ 58^′^N, 38^°^ 47^′^E. It has mean annual temperature of 15.9^°^C, total average precipitation 1089 mm and annual sunshine 2439 h. Fentale (Metehara) is one of the districts in the Oromiya Regional State, located in the Great Rift Valley, about 250 km East of Addis Ababa. The prevailing climate is arid. Most parts of this woreda range from 900 to 1000 m above sea level. Borena is located in Oromiya National Regional State, about 600 km South of Addis Ababa. The climate is generally semi-arid with annual average rainfalls ranging from 300 to > 700 mm (Oromiya Pastoralist Area Development Commission, OPADC 2012).

### Study camels

The main catchment source of these camels was mainly from pastoral livestock production systems of Borena and Fentale (Metehara) pastoralist areas. These areas possess large number of camels, Fentale (Metehara) with 61,425 camels and Borena with an estimated population of 174,185 camels (Oromiya Pastoralist Area Development Commission, OPADC 2012). The camels slaughtered at the abattoir were meant to meet consumer demand of Somali residents at Addis Ababa Bole Bulbula area where the camel meat sold at camel butcheries in that locality. On average, about five to eight camels were slaughtered on daily basis during the study period depending on the consumer demand of Somali residents at Addis Ababa Bole Bulbula. A total of 168 apparently healthy camels were considered to facilitate the reading of skin reaction after 72 h. Camels were carefully identified; age, sex, origin, and body condition score (BCS) were recorded. Body condition score was determined by hump structure according to previously established guideline indicated in the website: http://www.camelsaust.com.au/livebodycond.htm and the scores range from 1 to 5 and then categorized into three groups: poor, moderate, and good. Age category was determined by using the dental eruption and wear as previously described by Dioli and Schwartz (1992).

### Study design and sampling procedure

This study was abattoir-based cross-sectional study design to evaluate diagnostic test performance of the single intra-dermal comparative cervical tuberculin testing (SICCT) against gross pathology as reference diagnostic test to define disease status in camels. For sampling, the formula of random sampling of Thrusfield (2007) was employed. For this, 95% confidence level, 5% desired absolute precision, and expected prevalence of 10% (Mamo et al. 2011) was considered. Thus, a total of 168 apparently healthy camels were randomly selected based on the number of days of stay in the holding pen of the abattoir. Single intra-dermal comparative cervical tuberculin (SICCT) testing was conducted. Skin thickness before and after injection of the dose of tuberculin was recorded using graduated caliper. Data regarding skin tuberculin reactivity was recorded for individual camel. Tuberculin-tested camels were followed during post mortem examination, tissues from the lungs, lung-associated lymph nodes, and lymph nodes of the head were examined for the presence of any gross tubercle lesions. The lymph nodes (LN) were retropharyngeal LN, parotid, LN, sub-mandibular LN, trachea-brocheal, and cranial and caudal mediastinal LNs.

### Single intra-dermal comparative cervical tuberculin test (SICCT)

Tuberculin skin testing is the OIE standard test for the detection of bovine tuberculosis (OIE 2009). It involves intra dermal injection of bovine and avian tuberculin-purified protein derivative (PPD) and the subsequent detection of swelling (delayed hypersensitivity) at the site of injection 72 h later (OIE 2009). Single intra-dermal comparative cervical tuberculin (SICCT) testing was conducted using bovine and avian tuberculin-purified protein derivatives (PPD), injected intra-dermally with appropriate dose on the right side of the neck of the camels at 10-cm distance. Skin thickness before and after injection was recorded for individual camel using graduated caliper. Following the standard procedure, the skin at the middle of the right side of the neck of the camels were cleaned and shaved, the thickness were measured, the site was marked prior to injection, and the dose of tuberculin was then injected intra-dermally. The injection site was examined for swelling and thickness after 72 h. The difference between the skin reactivity to bovine PPD and avian PPD was recorded for each injection sites after 3 days (OIE 2008).

### Gross Pathology/Post mortem examination

Sub-mandibular, retropharyngeal, trachea-bronchial, cranial and caudal mediastinal, and mesenteric lymph nodes and including lung tissues were examined in detail during post-mortem examination under a bright-light source. The lobes of the left and right lungs were inspected and palpated externally. Then, each lobe was sectioned into about 2-cm thick slices to facilitate the detection of lesions with sterile surgical blades. Similarly, lymph nodes are sliced into thin sections (about 2-mm thick) and inspected for the presence of visible lesions. Whenever gross lesion suggestive of TB were detected in any of the tissue, the tissue was classified as having lesions and any evident gross lesions were recorded for each of the lymph modes examined. Camels with macroscopic lesions varying from firm or hard white, gray, or yellow nodule with a yellow, caseous, and necrotic center that was dry and solid to thin-walled suppurative abscesses were classified as post mortem positive as previously described by Mamo et al. (2009).

### Data analysis

Data were entered into Microsoft excel sheet, coded, and analyzed using statistical packages, STATA-version 13 and SPSS-version 20. Descriptive statistics were used to measure like tuberculin reactor and lesion detection rates, and chi-square test for associations of different variables. The receiver operating characteristics (ROC) analysis was employed for sensitivity and specificity evaluation of the SICCT at different cutoff points. For statistical significance, 95% confidence level and *P* value of 0.05 were considered.

## Results

Of the 168 camels considered to assess the performance of the SICCT at different cutoff points against gross pathology as a gold standard, positive tuberculin reactor rate with 95% confidence interval of [11.9% (11.6–12.23%)], [61.3% (60.9–61.8%)], [60.1% (59.6–60.6%)], [22.6% (22.2–23.02%)], and [22.6% (22.2–23.02%)] for the cutoff points ≥ 3.5, ≥ 1.5, ≥ 2.0, ≥ 2.5, and ≥ 3 mm, respectively. The frequency of positive reactors at different cutoff points of the SICCT and gross lesion detection rate in terms of host risk factors of camels were summarized in Table 1.

**Table 1:** Frequency of positive reactors and lesion detection rates in terms of host risk factors

Variable	No. (%) examined	Number (%) positive reactors of the SICCT at					No. (%) gross lesion
		≥ 2 mm	≥ 1.5 mm	≥ 2.5 mm	≥ 3 mm	≥ 3.5 mm	
Age							
≤ 7 years	42 (25)	30 (71.4)	30 (71.4)	9 (21.4)	9 (21.4)	6 (14.3)	6 (14.3)
> 7 years	126 (75)	71 (56.3)	73 (57.9	29 (23.0)	29 (23.0)	14 (11.1)	22 (17.5)
Sex							
Male	34 (20)	20 (58.8)	21 (61.8)	7 (20.6)	7 (20.6)	4 (11.8)	2 (5.9)
Female	134 (80)	81 (60.4)	82 (61.2)	31 (23.1)	31 (23.1)	16 (11.9)	26 (19.4)
BCS							
Poor	70 (42)	38 (54.3)	39 (55.7)	11 (15.7)	11 (15.7)	8 (11.4)	17 (24.3)
Moderate	50 (30)	36 (72)	37 (74)	18 (36)	18 (36)	8 (16)	5 (10)
Good	48 (28)	27 (56.3)	27 (56.3)	9 (18.8)	9 (18.8)	4 (8.3)	6 (12.5)
Origin							
Borena	121 (72)	75 (61.9)	77 (63.6)	29 (23.9)	29 (23.9)	14 (11.6)	21 (17.4)
Metehara	47 (28)	26 (55.3)	26 (55.3)	9 (19.1)	9 (19.1)	6 (12.8)	7 (14.9)
Total	168	101 (60.1)	103 (61.3)	38 (22.6)	38 (22.6)	20 (11.9)	28 (16.7)

The interpretation of the SICCT at different cutoff points revealed that the performance of SICCT at cutoff values ≥ 2.5, ≥ 3, and ≥ 3.5 mm were found to be better informative in discriminating diseased cases than the other cutoff values. Sensitivity of 60.7% and specificity of 85% for the cutoff values ≥ 2.5 and ≥ 3 mm; and sensitivity of 57.1% and specificity of 97% for the cutoff value ≥ 3.5 mm were recorded (Table 2). Hence, the use of a cutoff value ≥ 3 mm appeared to be appropriate in camels.

**Table 2:** The performance of the SICCT as compared to gross pathology

	Gross pathology to define true disease status			
SICCT (mm)	Se (%)	Sp (%)	*χ* ^2^	*p* value
≥ 2	71.4	41	1.45	0.16
≥ 1.5	68	41	0.84	0.24
≥ 2.5	60.7	85	27.9	0.000
≥ 3.5	57.1	97	65.6	0.000
≥ 3	60.7	85	27.9	0.000

**Fig. 1:**
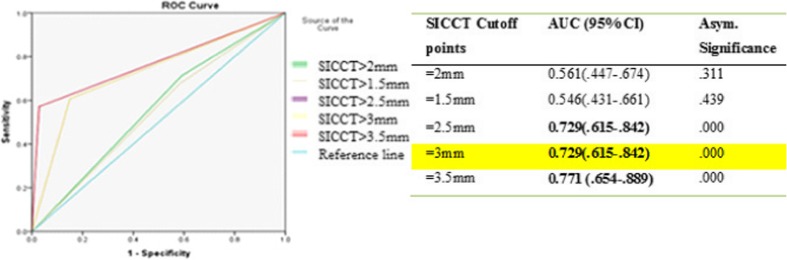
The receiver operating characteristics (ROC) analysis for the performance of the SICCT to detect tuberculosis in camels against gross pathology as a gold standard test

For the validity of the SICCT against gross pathology, the receiver operating characteristics (ROC) analysis revealed area under the curve (AUC) for cutoff point ≥ 3 mm was [0.729 (0.615–0.842)] and statistically significant (*P* = 0.000) for sensitivity and specificity evaluation (Fig. 1).

## Discussion

The performance of the SICCT to detect tuberculosis in camels revealed positive tuberculin reactor rates at different cutoff points suggesting tuberculosis is prevalent in dromedary camels. It was observed that gross visible lesions were detected in tuberculin reactor camels; however, non-tuberculin reactor camels were also detected with gross visible lesions. In the present study, variations in tuberculin reactivity and the degree of detection of gross tuberculosis lesions might be due to variations in sensitivity and specificity of diagnostic methods to discriminate disease status, the course of the disease itself where some camels might be at early stage while others might be with advanced stage of the disease to become non-reactors but with gross lesions. Moreover, the nature of tuberculin used, the degree of sensitivity of camels to bovine and/or avian tuberculin, the immune mechanism of camels, and the involvement of non-tuberculosis mycobacteria might be some other possibilities. Similar studies were previously conducted by different authors for dairy cattle at Addis Ababa, cattle in Central Ethiopia, and cattle in Cameroon, respectively (Ameni et al. 2008; Awah-Ndukum et al. 2012; Tsegaye et al. 2010). The use of different tests as a reference influences the outcome (sensitivity) and does not allow comparative assessment of various studies (Cousins and Florisson 2005).

The performance of the SICCT was found to be more informative at cutoff point ≥ 3 mm for sensitivity and specificity evaluation. In this study, better performance of the SICCT for dromedary camels in Ethiopia can be obtain if the cutoff value is lowered to ≥ 3 mm which would be of practical relevance to existing local conditions. Similar finding was reported by Awah-Ndukum et al. (2012) for cattle in Cameroon with better performance at cutoff values ≥ 3 and ≥ 3.5 mm. Lowering the cutoff value from the OIE standard has been recommended by different researchers for the African cattle for sensitivity and specificity evaluation (Ameni et al. 2008; Muller et al. 2009) which substantiate the present finding. The OIE-recommended cutoff value was established mainly in developed countries for *Bos taurus* cattle and different cutoff values are applied according to a particular country’s disease status and objective of its disease control program (Ameni et al. 2008; De la Rua-Domenech et al. 2006).

The ROC analysis for sensitivity and specificity evaluations of the SICCT at different cutoff points revealed severe interpretation at ≥ 3 mm cutoff point as compared to detection of gross lesion. The area under the curve of the ROC was significantly higher (*P* < 0.05) for this cutoff point. Thus, the sensitivity and specificity evaluation of the SICCT at the cutoff point ≥ 3 mm was found to be better informative in discriminating diseased case than the other cutoff points. Sensitivity (60.7%) obtained in this study at the cutoff point ≥ 3 mm was found to be lower than the sensitivity of 68.8% at ≥ 2 mm cutoff point for cattle in Ethiopia (Ameni et al. 2008), 67.8% at ≥ 3.5 mm for cattle in Cameroon (Awah-Ndukum et al. 2012) and lower than the sensitivity of 80% stated by OIE (2009) at ˃ 4 mm cutoff point. Various factors can influence the sensitivity of tuberculin skin test and the hypersensitivity reactions can fluctuate considerably depending on the animal. Delayed hypersensitivity reactions provoked by tuberculin injection can become established 3 to 6 weeks after exposure of the host to bacilli agents while recently infected animals may not react sufficiently to tuberculin injection. However, recently infected animals, animals under stress due to mal-nutrition, gastro-intestinal parasitoses, other concurrent infections, and animals with generalized Tb would be anergic and fail to react to tuberculin skin test. Thus, animals presenting the SICCT skin thickness of ≤ 4 mm should not be excluded that they are not affected by tuberculosis (Palmer 2006; Thoen 2009; Ameni et al. 2008; Awah-Ndukum et al. 2012; Inangolet et al. 2008; Ngandolo et al. 2009). Thus, the result of the present study could suggest the use of ≥ 3 mm cutoff value for the SICCT to diagnose TB in dromedary camels in Ethiopia.

## Conclusion

This study concluded that the SICCT if lowered from the OIE recommended cutoff ≥ 4 to ≥ 3 mm would be better informative for sensitivity and specificity evaluation and to detect tuberculosis in dromedary camels in Ethiopia. However, the detection of tuberculosis lesions and tuberculin skin reactions are not conclusive. Therefore, this study should be substantiated by further mycobacteriological culture and molecular tests.

## References

[CR1] Ameni G, Hewinson G, Aseffa A, Young D, Vordermeir M (2008). Appraisal of interpretation criteria for comparative intradermal tuberculin test for the diagnosis of bovine tuberculosis in Central Ethiopia. Clinical and Vaccine Immunology.

[CR2] Awah-Ndukum J, Temwa J, NguNgwa V, Mouiche MM, Iyawa D, Zoli PA (2012). Prevalence of bovine tuberculosis in cattle in the highlands of Cameroon based on the detection of lesions in slaughtered cattle and tuberculin skin tests of live cattle, Veterinari. Medicina.

[CR3] Beye AF, Zerom KG, Mussa A, Ameni G, Sanni MA (2014). Prevalence of bovine tuberculosis in dromedary camels and awareness of pastoralists about its zoonotic importance in Eastern Ethiopia. Journal of Veterinary Medicine and Animal Health.

[CR4] Cousins and Florisson (2005). A review of tests available for use in the diagnosis of tuberculosis in non-bovine species. Review science and technology of Office international Epizooties.

[CR5] de la Rua-Domenech R., Goodchild A.T., Vordermeier H.M., Hewinson R.G., Christiansen K.H., Clifton-Hadley R.S. (2006). Ante mortem diagnosis of tuberculosis in cattle: A review of the tuberculin tests, γ-interferon assay and other ancillary diagnostic techniques. Research in Veterinary Science.

[CR6] Dioli, M. and Schwartz, H.J., 1992. The one humped camel in eastern Africa: A pictorial guide to disease, Health care and management. Weikeersheini, Verlagiosef monograph, Pp. 1–59.

[CR7] Fowler Murray E., Bravo P. Walter (2013). Infectious Diseases. Medicine and Surgery of Camelids.

[CR8] Gumi B, Schelling E, Erenso G, Firdessa R, Biffa D, Assefa A, Tschopp R, Yimuah L, Young D, Zinsstag J (2012). Low Prevalence of bovine tuberculosis in Somali pastoral livestock, Southeast Ethiopia. Tropical Animal Health and Production.

[CR9] Inangolet FO, Biffa D, Oloya J, Opuda-Asibo J, Skjerve EA (2008). Cross-Sectional Study of Bovine Tuberculosis in the Transhumant and Agro-Pastoral Cattle Herds in the Border Areas of Katakwi and Moroto Districts, Uganda. Tropical Animal Health and Production.

[CR10] Jibril Yasmin, Mamo Gezahegne, Hanur Ibrahim, Zewude Aboma, Ameni Gobena (2016). Prevalence of camel tuberculosis and associated risk factors in camels slaughtered at Akaki Abattoir, Ethiopia. Ethiopian Veterinary Journal.

[CR11] Kassaye S, Molla W, Ameni G (2013). Prevalence of Camel Tuberculosis at Akaki abattoir in Addis Ababa, Ethiopia. African Journal of Microbiology Research.

[CR12] Mamo G, Kassaye A, Sanni M, Ameni G (2009). A cross sectional study of Camel Tuberculosis in Ethiopia. Bulletin of Animal Health and Production in Africa.

[CR13] Mamo G, Bayleyegn G, Sisay T, Legesse M, Medhin G, Bjune G, Abebe F, Ameni G (2011). Pathology of Camel Tuberculosis and Molecular Characterization of its Causative Agents in Patoral Regions of Ethiopia. PLOS: ONE, A peer-Reviewed Open Access. Journal.

[CR14] Muller B, Vounatsou P, Ngandolo BN, Diguimbaye-Djaïbe C, Schiller I, Marg-Haufe B (2009). Bayesian receiver operating characteristic estimation of multiple tests for diagnosis of bovine tuberculosis in Chadian cattle. PLoS ONE.

[CR15] Mustafa IE (2013). Bacterial diseases of dromedaries and bacterian camels. Review Science and technology of Office International Epizooties.

[CR16] Ngandolo BNR, Müller B, Diguimbaye C, Schiller I, Marg-Haufe B, Cagiola M (2009). Comparative assessment of fluorescence polarization and tuberculin skin testing for the diagnosis of bovine tuberculosis in Chadian cattle. Preventive Veterinary Medicine.

[CR17] Oromiya Pastoralist Area Development Commission (OPADC) (2012). Camel Development road map.

[CR18] Palmer MV (2006). Effects of Different Tuberculin Skin-Testing Regimens on Gamma Interferon and Antibody Responses in Cattle Experimentally Infected with Mycobacterium bovis. Clinical and Vaccine Immunology.

[CR19] Rhodes, S., Crawshaw, T., De la ReaDemenech, R., Bradford, S., Lyashchenko, K.P. and Mamo, G., 2015. Mycobacterial Infections in Camelids. In: Tuberculosis, Leprosy and Mycobacterial Diseases of Man and Animals, Pp. 216–234.

[CR20] Strain SAJ, James M, Stanley WJM (2011). Bovine tuberculosis: A review of diagnostic tests for M. bovis infection in cattle. Bacteriology Branch Veterinary Sciences Division. Agriculture Food and Biosciences Institute.

[CR21] Thoen CO (2009). Tuberculosis: A re-emerging disease of animals and humans. Veterinaria Italiana.

[CR22] Thrusfield, M., 2007. Veterinary epidemiology. 3rd ed. Royal (Dick) School of Veterinary Studies University of Edinburgh, Oxford, UK, Blackwell publishing company.

[CR23] Tsegaye W, Aseffa A, Mache A, Mengistu Y, Berg S, Ameni G (2010). Conventional and Molecular Epidemiology of Bovine Tuberculosis in Dairy Farms in Addis Ababa City. International Journal of Applied Research in Veterinary Medicine.

[CR24] Twomey D. F., Crawshaw T. R., Anscombe J. E., Barnett J. E. F., Farrant L., Evans L. J., McElligott W. S., Higgins R. J., Dean G. S., Vordermeier H. M., de la Rua-Domenech R. (2010). Assessment of antemortem tests used in the control of an outbreak of tuberculosis in llamas (Lama glama). Veterinary Record.

[CR25] World Health Organization (WHO), 2015. Global Tuberculosis report. 20^th^ edi, Geneva. Pp. 1–126.

[CR26] World Organization for Animal Health (OIE), 2008. Manual of Diagnostic Tests and Vaccines for Terrestrial Animals, http://www.oie.int/eng/normes/mmanual.htm

[CR27] World Organization for Animal Health (OIE), 2009. Manual of Diagnostic Tests and Vaccines for Terrestrial Animals. Camel tuberculosis: http://www.oie.int/eng/normes/mmanual.htm, Pp.1–16.

[CR28] Zerom K, Sisay T, Mamo G, Bayu Y, Ameni G (2013). Tuberculosis in dromedaries in Eastern Ethiopia. Abattoir-based prevalence and molecular typing of its causative agents in camels, Research. Journal.

